# Distinct trajectories of physical activity and related factors during the life course in the general population: a systematic review

**DOI:** 10.1186/s12889-019-6513-y

**Published:** 2019-03-06

**Authors:** Irinja Lounassalo, Kasper Salin, Anna Kankaanpää, Mirja Hirvensalo, Sanna Palomäki, Asko Tolvanen, Xiaolin Yang, Tuija H. Tammelin

**Affiliations:** 1 Faculty of Sport and Health Sciences, University of Jyväskylä, Jyväskylä, Finland; 2 LIKES Research Centre for Physical Activity and Health, Jyväskylä, Finland; 3 Methodology Center for Human Sciences, University of Jyväskylä, Jyväskylä, Finland

**Keywords:** Physical activity, Sport participation, Exercise, Longitudinal, Prospective, Trajectory, Finite mixture model, Review

## Abstract

**Background:**

In recent years, researchers have begun applying a trajectory approach to identify homogeneous subgroups of physical activity (PA) in heterogeneous populations. This study systematically reviewed the articles identifying longitudinal PA trajectory classes and the related factors (e.g., determinants, predictors, and outcomes) in the general population during different life phases.

**Methods:**

The included studies used finite mixture models for identifying trajectories of PA, exercise, or sport participation. Three electronic databases, PubMed (Medline), Web of Science, and CINAHL, were searched from the year 2000 to 13 February 2018. The study was conducted according to the PRISMA recommendations.

**Results:**

Twenty-seven articles were included and organized into three age group: youngest (eleven articles), middle (eight articles), and oldest (eight articles). The youngest group consisted mainly of youth, the middle group of adults and the oldest group of late middle-aged and older adults. Most commonly, three or four trajectory classes were reported. Several trajectories describing a decline in PA were reported, especially in the youngest group, whereas trajectories of consistently increasing PA were observed in the middle and oldest group. While the proportion of persistently physically inactive individuals increased with age, the proportion was relatively high at all ages. Generally, male gender, being Caucasian, non-smoking, having low television viewing time, higher socioeconomic status, no chronic illnesses, and family support for PA were associated either with persistent or increasing PA.

**Conclusions:**

The reviewed articles identified various PA subgroups, indicating that finite mixture modeling can yield new information on the complexity of PA behavior compared to studying population mean PA level only. The studies also provided novel information how different factors relate to changes in PA during life course. The recognition of the PA subgroups and their determinants is important for the more precise targeting of PA promotion and PA interventions.

**Trial registration:**

PROSPERO registration number: CRD42018088120.

**Electronic supplementary material:**

The online version of this article (10.1186/s12889-019-6513-y) contains supplementary material, which is available to authorized users.

## Background

Up to four-fifths of adolescents and one-third of adults do not meet the public health physical activity (PA) recommendations [1]. The high prevalence of physical inactivity imposes a substantial economic burden on health care resources [2, 3]. For these reasons, the promotion of PA should be a standalone public health priority [4]. Studying PA behavior and its correlates longitudinally can help better understand how PA develops and why during the life course.

Previous research has established that overall PA decreases with age and is lower among women [1, 5]. Longitudinal data show that an individual’s earlier PA predicts similar PA behavior in the future [6–8], and that PA tracks at low to moderate levels from childhood to adulthood [9]. While tracking shows whether individuals maintain their PA ranking in the study population over time [10], it does not necessarily address the issue of whether they maintain their PA level. As for trajectory approach, it allows study of the development of behavior (e.g., PA level) over time [11, 12]. A trajectory describes the development (stability or change) in an individual’s behavior over a relatively long period [11, 13]. Whereas change in time is traditionally studied with two measurement points, trajectory studies use multiple measurement points enabling the study of linear as well as curvilinear change in time [14]. This means that the longitudinal nature of the data can be exploited more thoroughly, and the variation in the magnitude, rate and timing of possible change can be studied more precisely.

While standard growth curve modeling has been applied for studying the mean PA trajectory of a population and how individual variation about that mean relates to predictors [15–17], not until recent years, the number of studies identifying distinct PA trajectory classes (i.e., subgroups) has increased. In these studies, the target behavior of individuals in the same trajectory class is expected to be similar, while it differs from that of the individuals in the other classes [14, 18]. In addition, it is possible to study how potential predictors, determinants and outcomes relate to the distinct trajectories [11, 13, 18, 19]. Identifying distinct PA trajectories at different phases of life as well as examining the factors related to the trajectories is important for planning tailored and well-targeted PA promotion strategies and interventions – especially for those who are inactive or at risk of becoming inactive.

Trajectory classes have been identified using various statistical approaches, such as latent class analyses, latent class growth analyses, growth mixture modeling and group-based trajectory modeling [11, 12, 18–20]. These are all known as finite mixture modeling methods [19, 20]. The strength of finite mixture modeling is that rather than assuming the existence of distinct trajectories in a population, it identifies them based on the population data [11, 13, 14]. Thus, in finite mixture models the number of underlying trajectories, their shape, their prevalence in the population and the assignment of individuals to them are all inferred from the data [12, 20].

However, data on PA trajectories identified in different study populations and the factors that explain why certain individuals follow certain PA trajectories have not yet been assembled. This paper systematically reviews the longitudinal studies that have used finite mixture modeling to identify distinctive PA trajectory classes in the general population at different life phases. The aim is to provide an overview of the research on trajectory classes by gathering and categorizing the data reported on the shapes, proportions and descriptions of trajectory classes, taking into consideration the age of the participants. In addition, the review investigates potential factors (e.g., determinants, predictors and outcomes) associated with specific PA trajectories.

## Methods

### Protocol and registration

This systematic review was conducted following the Preferred Reporting Items for Systematic Reviews and Meta-Analyses (PRISMA) statement [21], and applying the PRISMA checklist (Additional file 1: Table S1). The review protocol was registered with the International Prospective Register of Systematic Reviews (PROSPERO) and is available from http://www.crd.york.ac.uk/PROSPERO/display_record.php?ID=CRD42018088120 (Registration number: 2018: CRD42018088120).

### Search strategy and data sources

Since preliminary searches showed that no articles identifying distinct PA trajectories had been published before the year 2000, the search was conducted from the year 2000 to 13 February 2018. The following databases were systematically searched: PubMed (Medline), Web of Science, and CINAHL. To ensure a comprehensive coverage, a wide range of search terms was used. To be eligible for consideration, articles had to contain a mention of the term *trajectory* and, for each of the following fields, at least one of the italicized terms: (a) for PA: *physical activity, physical inactivity, sport, exercise* or *team participation*; (b) for the study design: *longitudinal, cohort, prospective, panel* or *follow-up;* and (c) for distinct trajectory classes: *group, cluster, class, profile, subgroup* or *classification.* For details on the search strategy, see Additional file 2: Table S2.

### Inclusion and exclusion criteria

To be included in the review, studies had to (a) be longitudinal in design; (b) report data on children, adolescents, adults or older adults in the general population; (c) identify more than one trajectory class with three or more measurement points for either PA, exercise or sport participation (SP); (d) use finite mixture modeling for the identification of trajectories; (e) be published between the year 2000 and 13 February, 2018; and (f) be published in English.

As the purpose of the review was to identify studies specifically using finite mixture modeling, studies using statistical methods where the number of classes was decided a priori or methods that assumed the individuals come from a single population that can be described and approximated adequately with a single growth trajectory (e.g., growth curve modeling), were excluded. Also excluded were studies combining other variables (e.g., other health behaviors) with PA in the same trajectory modeling, conference abstracts without existence of published article, and studies confined to groups with significant co-morbidities (e.g., chronic illnesses, disabilities or mental health problems) or that solely concerned pregnant women.

### Study selection process

To establish inter-rater reliability, studies were selected by two authors (IL and KS) independently. First, the titles and abstracts of candidate articles were screened for relevance. After a relevant abstract was found, the full text was assessed for eligibility. Where uncertainties arose about the inclusion of an article, the other authors (AK, MH, AT, and SP) were consulted.

### Data extraction

When available, the following data were extracted from each article: reference (authors and publication year), study aims, description of the study (name of the study, geographical location, study design, year of the baseline measurement, follow-up time, and number of measurements), study sample (sample size, distribution of males and females, and age at baseline), statistical approach (the finite mixture model used to identify the trajectory classes, the software used for the analysis, the criteria used for model comparison, determining the final number of trajectory classes, and the goodness of fit of the model), data collection and PA variables, description of the trajectory classes identified (the number and names of the trajectory classes and the proportion of participants in each trajectory class), factors related to the PA trajectories (predictors, determinants, outcomes, and covariates), and main findings in relation to trajectory class membership. One author (IL) performed the data extraction, consulting other authors when needed (MH, AK, KS, THT, and SP).

### Quality assessment

Two authors (IL and MH) independently assessed the methodological quality of the included studies. Any disagreement or uncertainty was resolved by consulting a third author (AK). Study quality was assessed by using modified versions of the Quality Assessment Tool for Observational Cohort and Cross-Sectional Studies (https://www.nhlbi.nih.gov/health-topics/study-quality-assessment-tools) and Guidelines for Reporting on Latent Trajectory Studies (GRoLTS) -checklist [22]. The GRoLTS-checklist is mainly aimed for studies using latent growth mixture modeling and latent class growth analysis, but when modified, is usable for all finite mixture models. The researchers also included some additional criteria for example, objective vs. self-reported measures of PA, whether more than one statistical fit measure was used when deciding the final number of trajectory classes and whether at least one of the fit measures was the Bayesian Information Criterion (BIC) or adjusted Bayesian Information Criterion (ABIC). These criteria were added in response to the recommendation that researchers use more than one comparison tool for deciding the final number of trajectory classes and that the BIC or ABIC should be one of them [22]. The questions chosen for the quality assessment were aimed to assess attrition bias, performance bias, selection bias concerning the final number of trajectory classes chosen, detection bias, and other biases (e.g., were covariates used).

Each included study was rated in quality from zero to 16. The study under assessment was marked “yes” when it met a given quality criterion and “no” when it did not, “NA” when the criterion was not applicable, and “CA” when meeting the criterion could not be determined from the information provided by the study. In addition, if a specific quality criterion was not mentioned in the assessed study but reference was made to previous studies where the criterion was mentioned, it was marked “yes”. Studies scored 1 point for each yes and 0 points for each no, “NA” and “CA”. Study quality was categorized as poor (score ranging from 0 to 5), fair (6 to 11), or good (12 to 16).

### Analyses

To summarize the PA trajectory classes identified in the general populations studied and the factors explaining these classes, the relevant data were first extracted from the included articles and a qualitative synthesis, including tables and figures, was performed. The articles were organized into groups based on the participants’ age in each study. Each of the PA trajectories reported in the studies was subsumed under a PA trajectory category that best described the name and shape of the trajectory. To illustrate the proportion of participants within a trajectory class (i.e., class size of the trajectory) and to compare this to the proportions in the other trajectory classes within and between the PA trajectory categories (*p*), a forest plot was generated. For the forest plot, standard errors (SE) and 95% confidence intervals (95% CI) for the class sizes reported in the individual studies were calculated using the formula SE =$\sqrt{p\left(1-p\right)/n}$, where *n* is the sample size of a study; 95% CI: *p ±* 1.96 SE. The forest plot was generated using R version 3.2.2 (package forestplot). The results could not be combined in a quantitative synthesis (i.e., meta-analysis) as the inclusion criteria were based on diverse PA, exercise and SP variables and the participants were examined at different phases of life in different studies. The mean age at which PA level started to decrease among children and adolescents was calculated using IBM SPSS Statistics (version 24).

## Results

### Overview of the included studies

**Fig. 1:**
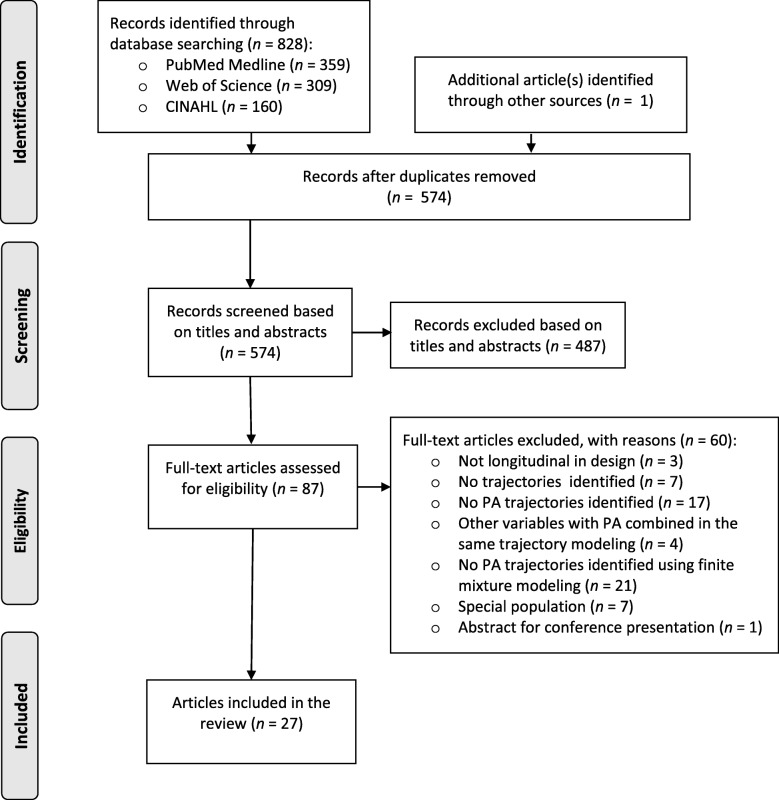
PRISMA flow chart for the selection of studies. *Abbreviations:* PRISMA = preferred reporting items for systematic reviews and meta-analyses; PA = physical activity

The literature search identified 828 articles and an additional study [23] was added through other sources. After discarding duplicates, 574 potential articles remained. After screening the titles, abstracts, and full texts of the articles independently, the two authors (IL and KS) found that they had each selected the same 27 articles and two different articles each that potentially met the inclusion criteria. Full consensus was reached after discussion and 27 papers were deemed to have fully met the inclusion criteria (Fig. 1; Additional file 3: Table S3). All studies were published between years 2004 and 2018.

Objectively measured PA was available from three different research data and was used in six studies [24–29] while the others used self-reported or parent-reported measures of PA [30–45], exercise [46, 47] and SP [23, 26, 28, 48, 49] (Additional file 3: Table S3). The broader term PA trajectories is used to refer to all the different trajectories of PA, exercise and SP. Specific information is provided separately for PA and SP only for the qualitative synthesis of trajectories during childhood and adolescence and also in Additional file 4.

The number of PA trajectory classes identified in each study ranged from one to five, and was most commonly either four [23, 24, 26–28, 31, 36, 37, 39–41, 46] or three [24–26, 28, 29, 33–35, 42–45, 48, 49] (Additional file 3: Table S3). Eight studies identified separate trajectories for males and females [24, 25, 38, 43, 44, 46, 48, 49], twelve studied males and females together in the same trajectories [23, 26–28, 30–32, 36, 40–42, 45], five studied females only [29, 35, 37, 39, 47], and two studied males only [33, 34].

### Overview of study quality and risk of bias

The quality assessment results are shown in detail as supplementary material (Additional file 5: Table S4). Initial agreement on study quality between the two raters (IL and MH) was 90%, and full consensus was reached after discussion. Study quality scores ranged from 9/16 to 15/16 (mean = 12). None of the 27 studies were rated as of poor quality, 11/27 were rated as fair (scores 9: n = 3 [43, 44, 48]; 10: n = 3 [34, 37, 46]; 11: n = 5 [24, 25, 31, 41, 47]), and 16/27 as good (scores 12: n = 3 [38, 40, 45]; 13: n = 2 [23, 32]; 14: n = 3 [36, 42, 49]; 15: n = 8 [26–30, 33, 35, 39]). Frequent reporting on items from the modified Quality Assessment Tool for Observational Cohort and Cross-Sectional Studies was observed [see Additional file 5: Table S4; questions 1–9]. However, reporting on items from the modified GRoLTS-checklist was more infrequent: only ten of the 27 studies reported on all of the six items selected from the GRoLTS-checklist [see Additional file 5: Table S4; questions 11–16] while seven studies reported on just one and three on two items.

Nineteen distinct longitudinal research data were used in the 27 articles (Additional file 3: Table S3). To avoid the reporting of duplicated results, the exact same PA trajectories derived from the same data are treated as one and the same result instead of as separate results [26–28, 43, 44]. The potentially different outcomes, determinants or predictors of all trajectories are still reported separately for each study. Nevertheless, since the review aimed to capture all the different PA trajectory classes thus far identified in general populations, when the shape, class size or the number of PA trajectories identified differed between studies, they were treated as separate results, including when derived from the exact same data.

### The age groups and PA trajectory categories derived from the reviewed studies

The studies were organized into three groups based on the participant age: youngest, middle, and oldest (Additional file 3: Table S3; Additional file 4). The youngest group consisted of children, adolescents and young adults, while the oldest group consisted of late middle-aged and older adults. The middle group was mixed, consisting largely of adults with the study period initiated in childhood, adolescence or adulthood and, in some studies, continuing up to old age.

Each PA trajectory reported in the included studies was classified under a PA trajectory category that best described its name, shape, and context (Additional file 4). The middle and oldest groups were distributed across six PA trajectory categories: Increaser, Highly active, Active, Inactive, Decreaser from a moderate PA level, and Decreaser from a low PA level / low-active. In the youngest group, an additional category, labelled Decreaser from a high PA level, was detected, and the Increaser category was replaced with a category labelled From increaser to decreaser. The purpose of the forest plot (Additional file 4) is to provide a comprehensive visualization of the prevalence and class sizes of distinct trajectories in general population at different phases of life. It also summarizes in one figure the sizes of the study samples and the PA measures and data collection method used in each study.

### PA trajectories in the youngest group

The youngest group contained 11 articles [23–28, 30, 38, 39, 48, 49] (Additional file 3: Table S3). Participants were aged four to 14 at baseline with the oldest participants aged 22 at the end of the latest follow-up. Follow-up time varied from two to 15 years (mean = 9.3 years; median = 8 years) and sample size from 530 to 8978 participants between studies.

In most studies, the trajectories with the highest proportion of individuals (range 39–100%) were placed in the following PA trajectory categories: Decreaser from a moderate PA level [24, 28, 39], Active [23, 28, 38, 49] or Decreaser from a low PA level / low-active [24, 30, 48] (Additional file 4: c, f, g). Altogether, 53 different PA trajectories were reported in the studies in the youngest group (Additional file 4). Persistent PA was described only in 14 trajectories: 11 in the Active category and three in the Highly active category (Additional file 4: b, c). Analysis of the shapes of the distinct trajectory classes showed that inactivity and low activity were more persistent behaviors [23, 28, 30, 38, 49] than high or moderate activity [23–25, 28, 30, 39, 49], meaning that those following the high or moderate PA trajectories more often showed change (usually a declining change), while the inactives remained at the same level.

More than half of the 53 trajectories described a decline in PA level during childhood and adolescence (Additional file 4: a, e, f, g). In fact, no studies found clear trajectories of continuously increasing PA: even an initially ascending trajectory was followed by a descent (Additional file 4: a). The declining trend in PA was especially clear in studies using objectively measured PA, where as many as 18 of the 21 trajectories described a decline in PA level [24, 25, 28]. Mean age at the start of the decline was 7.7 years in the studies using objective measures [24, 25, 28] compared to 9.6 years in those using self-reports of PA [32, 38, 39] or 10.8 years in those using self-reports of SP [28, 48, 49]. Although decreasing trends were prevalent in the youngest group, the PA level of the decreasers from high or moderate PA did not usually fall to the level of the inactive individuals [24, 30, 38, 48]. However, one study using objectives measures showed that the 95% CIs of all the three declining PA trajectory classes overlapped by the age 17 for girls [25]. Additionally, drop-outs from SP had lower SP rate in young adulthood than those who were initially identified in the non-participation trajectories [28, 49].

### PA trajectories in the middle group

The middle group comprised 8 studies [29, 32, 40–45] (Additional file 3: Table 3). Participant age at baseline ranged from 9 to 90. Two studies using the same data identified trajectories from childhood to adulthood [32, 42], another two, using different data, identified trajectories from adolescence to adulthood [43, 44], one studied adults only [45], and the other three studied adults and older adults [29, 40, 41]. Follow-up time varied from two to 34 years (mean = 19.8 years; median = 23.5 years), and sample size from 497 to 3564 participants.

Altogether 28 different middle group PA trajectories were reported in the studies (Additional file 4). In most studies, the largest proportions (range 43–86%) of individuals were in the trajectory classes describing either persistent PA [42–44], inactivity [40] or low PA [29, 32, 45] (Additional file 4: c, d, g). Stable PA trajectories (22 trajectories) were more prevalent than change (i.e., a decrease or increase in PA level) trajectories (6 trajectories) (Additional file 4). In the middle group, the one study measuring PA objectively identified three distinct but stable PA trajectories among women verifying the observation that PA seems to be stable behavior in adulthood [29].

Unlike in the youngest group, three trajectory classes indicating a continuous increase in PA level were reported [32, 40, 41]. One study that identified an increasing PA trajectory from adolescence to adulthood showed that the increasers had very similar PA level as the low-active and inactive participants until the age 12. However, by the age 15 the increasing trajectory began to differ from the low and inactive trajectories with the increase in PA level continuing into adulthood [32]. In two studies, the decreasing PA trajectories did not fall to the level of the inactive trajectories [32, 41] and in one study it did [40].

### PA trajectories in the oldest group

Eight studies were placed in the oldest group [31, 33–37, 46, 47] (Additional file 3: Table S3). Age at baseline was 35–55 in one article [31], 40 or older in two articles [33, 47], 50 or older in three articles [35, 36, 46], and 60 or older in two articles [34, 37]. Follow-up time varied from seven to 20 years (mean = 13.9 years; median = 12.0 years) and sample size from 433 to 92629 participants.

Altogether 34 different PA trajectories were reported by the studies in the oldest group (Additional file 4). While seven out of eight studies identified a trajectory describing persistent PA (range 14–51%) (Additional file 4: c), in five studies the highest proportion of individuals (range 26–72%) were in the inactive (Additional file 4: d) [36, 37, 46] and low-active trajectory classes (Additional file 4: g) [35, 47]. In addition, the PA level of those following the decreasing PA trajectories often fell to the level of those following the inactive trajectories [31, 36, 37, 46]. Some studies detected a declining trend in PA level with age, not only in one, but in several trajectory classes [34, 37]. These results show the prevalence of inactivity in older age and how PA levels tend to decline with age. Five studies, however, identified a trajectory class of increasers as well (Additional file 4: a) [31, 33, 36, 46, 47].

### Factors related to the PA trajectories

Most studies also aimed to explore what potential factors (i.e., predictors, determinants, and outcomes) are associated with specific PA trajectories. The most commonly studied factors were socioeconomic status (SES), family or social support, sociodemographic characteristics, health behaviors, and health-related variables (Additional file 3: Table S3).

Several studies in all three age groups showed that either higher education, higher income or higher occupational status was associated with a higher probability of following an active rather than inactive trajectory [26, 31–33, 35, 36, 38, 40, 46–48]. Having children was associated with decreasing PA in early midlife [32] while having parental PA support [32] or having a physically active father was an important factor, especially in lower SES families, in the adoption of a physically active lifestyle by a child [26].

Gender differences were observed in PA trajectory membership: active trajectories were more prevalent among males than females [24, 25, 28, 31, 38, 40, 46], just as participation in sport was more frequent among boys than girls [48, 49]. In contrast, the studies using data from Finnish cohorts found no marked gender differences in the membership of the different trajectory classes [43, 44]. Additionally, while Finnish males had higher odds of being persistently active, they also had higher odds of being persistently inactive rather than low active when compared to women [32]. Being non-Caucasian was a risk factor for low PA level [23, 35, 39, 47].

Associations of PA trajectories with other health behaviors were studied in all age groups. Current smoking was associated with decreasing PA, persistently low PA, or inactivity rather than with persistent PA [23, 30, 32, 33, 35, 47], and smoking cessation with increased levels of PA [33]. Greater alcohol consumption was associated with persistent PA [32, 35] and even with increasing PA [33]. Additionally, consistent PA was associated with decreasing television viewing time in adolescence [28, 39]. Older participants with lower fat intake and higher consumption of vegetables and fruits were more likely to follow a physically active than minimally active trajectory [35]. Over-weight and obesity were associated with low activity or inactivity among adult women [29] and older adults [33, 35, 37, 47]. A decreasing PA level in childhood [27] and low SP in adolescence [49] predicted unfavorable changes in body composition in young adulthood while persistent PA in childhood and adolescence predicted a better health profile [49] and greater bone strength [25].

Several different health-related factors were studied, especially among older adults. Older adults with diagnosed chronic diseases (e.g., arthritis, arthrosis, bronchitis, coronary artery disease, chronic obstructive pulmonary disease, or high blood pressure) [33, 35, 37, 47], or who had physical difficulties [47], disabilities [37], depressive symptoms [37, 46], or fair or poor self-rated health [37, 46] were less likely to follow a persistently active trajectory and more likely to follow a low-active or inactive trajectory. In addition, a low mortality rate [37] and good physical functioning [47] were associated with persistent PA. Depressive symptoms were also studied among youth and younger adults. Boys who dropped out from organized sport had higher depression scores at age 20 when compared to consistent sport participants [49]. However, Kaseva et al. found no association between leisure time PA from childhood to adulthood and the progression of depressive symptoms in adulthood.

## Discussion

To the best of our knowledge, this systematic review is the first to compile the literature on studies using finite mixture modeling to identify distinct trajectory classes of either stable or changing PA, exercise or SP in the general population during different life phases along with the examination of the potential factors related to these trajectories. The number of studies has started to accumulate: of the 27 included articles, 24 were published from 2010 onward. This reflects the novelty and topicality of the research area. The most common number of trajectory classes reported was three or four (Additional file 3: Table S3). The fact that several PA trajectory classes were identified shows that PA is a behavior that does not develop uniformly between individuals. This is why finite mixture modeling is an appropriate method for studying PA across the life course. Various distinct decreasing PA trajectories were reported among youth, in particular, while among adults and older adults few studies found increasing PA trajectories. The results were in agreement with previous findings showing that the proportion of inactive individuals is rather high at all ages and that inactivity tends to increase with age [1]. Various factors explaining the differences in PA level between individuals during the life course were studied, the strongest associations being found for SES and gender.

### Developmental trajectories of PA during the life course

The number of distinct PA trajectories describing change were more prevalent among youth, while persistently stable PA trajectory classes were more prevalent in adulthood. The inactive trajectories seemed to be more stable than the trajectories describing activity. These findings support research showing that low activity and inactivity track better than activity, and that the stability of tracking is higher during adulthood than in childhood or during the transition from childhood to adolescence or from adolescence to adulthood [9]. However, the trajectory studies reviewed here add to these findings by showing what specific changes in PA level occur between individuals during the life course.

All the reviewed studies in the youngest group identified at least one distinct PA trajectory describing a decrease in a high, moderate or low level of PA, a curvilinear trajectory describing an increase followed by a decrease in PA [24, 25, 28, 30, 38, 39], or drop-out from SP [23, 28, 48, 49]. This result is in line with previous findings showing that childhood [50, 51] and adolescence [52–54] are periods of life characterized by a decline in PA. Interestingly, the reviewed studies using objective measures of PA found that the level of PA had already started to decline at the age of school entry [24, 25, 28] whereas the studies using self-reported measures of PA found the corresponding age to be around 10 years [32, 38, 39]. It should be pointed out that some of the studies using self-reported measures studied slightly older children than the studies using objective measures, a factor that could partially explain this difference. However, regardless of measurement type, the trajectory studies showed that PA starts to decline in childhood, and not in adolescence – an observation also emphasized in other reports based on objectively measured PA [51, 55]. However, drop-out from SP might be more common in adolescence than in childhood since the age at which the decline began was higher in the SP than PA trajectories [23, 28, 48, 49].

While no trajectory classes characterized by consistently increasing PA were observed in the youngest group, such classes were reported in few of the studies in the middle [32, 40, 41] and oldest [31, 33, 36, 46, 47] groups. In the oldest group, the participants were younger in the five studies identifying a trajectory of increasing PA [31, 33, 36, 46, 47] than those in the three studies reporting no such trajectories [34, 35, 37]. Nguyen et al. [35] found a minimal but significant increase in PA over time in their moderately and highly active trajectory classes among 50- to 69-year-old women whereas a slight decrease was observed in PA in each parameter over time among the oldest participants (70 years or older). Various explanations have been offered for this. It has been suggested that adults increase their PA level due to aging-related health concerns [56, 57] or after retirement [58–60], while overall PA tends to eventually decline with older age [1], possibly due to declining health [60]. In most of the reviewed studies examining older adults [31, 36, 37, 46], those in the declining PA trajectories eventually approximated to the PA level of those in the inactive trajectories [31, 36, 37, 46]. At the same time, the declining trajectories usually did not fall to the level of the inactive trajectories in the studies examining children and adolescents [24, 30, 38, 48] or adults [32, 41]. Thus, despite of the common declining tendency of PA throughout life course, being physically active in childhood and adolescence may be of high importance since it can postpone the time of becoming inactive later on.

### Factors related to PA trajectory class membership

Various predictors, determinants, covariates and outcomes of PA trajectory class membership were studied. Mostly, these findings further supported other findings that have shown how: (1) higher SES is associated with PA in youth [61–63] and in adulthood [55]; (2) having family support [54, 61], active parents [61, 64], and especially an active father [63, 65], is associated with PA in childhood; and (3) males are generally more active than females [1, 54, 56, 64, 66]. One gender-related exception was found in studies on the Finnish population: leisure time PA was as common among adult women as among men [32, 43], a finding that has also been reported in a study not included in this review [67]. Most of the present results [33, 35, 37, 46, 47, 49] also further supported other findings showing that lack of PA is a major risk factor for morbidity and premature mortality [68–70]. However, one reviewed study found that Caucasian participants in a trajectory labeled “exceeding PA guidelines three times” had higher odds for developing subclinical coronary artery disease by middle age than those in the trajectory “below PA guidelines” [45], suggesting that extremely high doses of leisure time PA might be a risk factor for cardiovascular health.

Thus, most of the present results specifically supported other findings on correlates of persistent inactivity or persistent PA. Apart from studying stable PA trajectories, finite mixture modeling has the advantage of detecting changes over time enabling also the study of factors associated with these changes. The reviewed [23, 30, 32, 33, 35, 47] and other studies [71, 72] have found a negative association between regular smoking and PA with the reviewed studies showing that smoking cessation [33] and non-smoking [32] were associated with increasing PA, whereas an increase in smoking was associated with decreasing PA [30]. While the association between television viewing time and PA has been found to be negative and rather small [73, 74], the present trajectory studies add to this finding by suggesting that persistent PA is associated with decreasing television viewing time [28, 39] whereas decreasing PA is associated with increasing television viewing time [39] in adolescence. Moreover, alcohol consumption was positively associated with both increasing [32, 33] and decreasing PA trajectories [32] rather than with persistently low PA. Rovio et al. [32] also found that having children was associated with membership of a decreasing PA trajectory, an association also observed elsewhere [75]. Groups disadvantaged with respect to education and income were significantly more likely to be on a decreasing than active trajectory [40], while high adulthood education was associated with membership of both increasing and decreasing active trajectories [32]. Special attention should be paid to success at school and parental PA support in childhood since they were both associated with membership of a consistently increasing PA trajectory which began to differ from the inactive and low-active trajectories after the age of 12 [32].

### Limitations

This review also has its limitations that could induce bias in interpretation of the results. Gathering and comparing the findings was challenging due to the heterogeneity in study populations, sample sizes, follow-up duration, measurement times, time between measurements, participants’ age, the names researchers gave to their trajectories, measurements (PA, exercise, and SP), exposures and outcome variables, data organization in trajectory modeling (i.e., age vs. measurement year), and the finite mixture models used. For example, if the population in one study had lower SES than the populations in other studies, it could be expected to contain a larger group of inactive individuals. The length of the follow-up and the time between measurement points have been found to affect the number of trajectories identified [22], for example, a long interval between measurement points might mean that some PA patterns are not detected. Also, since the trajectory classes were usually labelled in relation to the other trajectory classes identified within each study and not necessarily in relation to PA guidelines, it is possible, for example, that a PA level reported as high in one study might be reported as moderate in another study.

Although articles reporting findings based on the exact same trajectories were omitted, a risk of reporting partially overlapping results remains when PA trajectories were initially identified by gender and then again for both sexes combined [25, 28, 36, 46], or when studies used the same data and variables but diverged over the final number of trajectory classes [32, 42]. The explanation for the latter case might be that Kaseva et al. [42] used Akaike’s Information Criterion (AIC) indices, despite the current recommendation that the BIC and ABIC are the best model fit measures for determining the final number of classes compared to, for example, the AIC or Lo-Mendel-Rubin-likelihood ratio test [22]. There is a possibility for selective bias in the final number of trajectory classes when the researchers estimated the shape of the trajectory only up to quadratic shape in studies having more than three measurement points [24, 28, 36–39, 41, 42, 48] which would also enable the estimation of, for example, cubic shape. The possibility of selective bias also exists when classes containing less than 5% of the study population were ignored [30, 33]. Other factors relating to bias at the individual study level are listed point by point in the additional material (Additional file 5: Table S4).

While finite mixture modeling has its advantages, presented in the beginning of this review [11, 12, 22], the developers of the modeling also have recognized the uncertainties related to these models, and a few studies have recommended caution when using them [20, 76–78]. For example, the correct assignment of individuals to a trajectory class cannot be certain, the number of trajectory classes is not immutable [76], and the choice of the optimal number of classes is based on various formal and informal criteria [20]. Thus, while model fit indices (e.g., BIC and ABIC) are available for determining the optimal number of classes, researchers also rely on the share of cases assigned to the smallest trajectory class, interpretability, and model convergence [19, 20]. The observations of this review support these comments that no consensus has not yet been achieved in the use of statistical approaches for identifying developmental trajectories [20, 78] and that the selected statistical approach itself may have an effect on the results [20]. The GRoLTS-checklist has been developed to address these uncertainties; even so, researchers should be alert to new developments in this rapidly evolving area [22].

### Future studies

Physical inactivity is the fourth leading risk factor for mortality worldwide [70]. To counter inactivity, future research should pay special attention to identifying additional determinants of trajectory class membership (e.g., social capital, environmental, psychological and genetic factors, cultural and social norms, global media and marketing, urbanization, sleeping, dietary behavior, and other life changes). Special attention should be paid to those who increase their PA, as it is important to understand how potential lifelong inactivity could be reversed to activity. The lack of longitudinal trajectory studies on the transition from adolescence to adulthood needs to be addressed to more profoundly. In addition, more large-scale population-based longitudinal studies using objective measures for identifying PA trajectory classes are needed as objectively measured and self-reported PA have shown only modest agreement [79]. Since all the reviewed studies were conducted in high-income countries (in Europe, USA, Canada, Australia or Taiwan), there is a need to identify PA trajectories in low- and middle-income countries. Finally, specification of the most suitable statistical model for identifying PA trajectories would help in collectively building knowledge in this field. Before this is achieved, use of the GRoLTS-checklist [22] is recommended in future trajectory studies using finite mixture modeling for standardizing the results.

## Conclusions

This review supports earlier findings on the general trends and population means of PA during the life course. However, studying diverse trajectory classes using finite mixture modeling also adds to previous knowledge by providing evidence of the heterogeneity of PA. Thus, this review broadens understanding of the variation that occurs in the timing, rate and magnitude of PA development during the life course and the factors relating to membership of specific trajectory classes. This helps identify the key periods of life and key groups at which to target PA promotion and thereby contribute to improving public health. The results showed that PA begins to decline already in childhood, that the PA habits stabilize with age and that inactivity is more persistent than activity. Hence, PA interventions should be targeted at children early in life before their PA habits become stable. Due to the heterogeneity in samples, finite mixture models, exposures and outcome variables used in the selected studies, and the general reliance on self-reported PA, the results of this review need to be interpreted cautiously.

## Additional files

Additional file 1: **Table S1.** PRISMA checklist. (PDF 304 kb)

Additional file 2: **Table S2.** Details of the search strategy. (PDF 247 kb)

Additional file 3: **Table S3.** Characteristics of the included studies, physical activity trajectories and related factors reported, and main findings in each study. (PDF 462 kb)

Additional file 4: **Forest plot.** Physical activity trajectories divided into categories by age group. (PDF 264 kb)

Additional file 5: **Table S4.** Quality assessment checklist and quality scores of the included studies. (PDF 180 kb)

## Abbreviations

ABIC: Adjusted Bayesian information criterion
AIC: Akaike’s information criterion
BIC: Bayesian information criterion
CI: Confidence interval
GRoLTS: Guidelines for reporting on latent trajectory studies
n: Number of cases
PA: Physical activity
PRISMA: Preferred reporting items for systematic reviews and meta-analyses
PROSPERO: International prospective register of systematic reviews
SE: Standard error
SES: Socio-economic status
SP: Sport participation
