# Supplementary material for: Distinct trajectories of physical activity and related factors during the life course in the general population: a systematic review
Source: BMC Public Health. 2019 Mar 6;19:271. doi: 10.1186/s12889-019-6513-y (PMC6404287; doi:10.1186/s12889-019-6513-y)
Supplement: Supplementary file 2 — Table S2. Details of the search strategy. (PDF 247 kb) [file 12889_2019_6513_MOESM2_ESM.pdf]

Additional file 2: Table S2. Details of the search strategy.

#	Searches
1.	trajector*
2.	physical activit*
3.	physical inactivit*
4.	sport
5.	sports
6	exercise
7.	exercises
8.	team participation
9.	2 or 3 or 4 or 5 or 6 or 7 or 8
10.	longitudinal
11.	cohort
12.	prospective
13.	panel
14.	follow-up
15.	follow up
16.	10 or 11 or 12 or 13 or 14 or 15
17.	group
18.	groups
19.	cluster
20.	clusters
21.	class
22.	classes
23.	profile
24.	profiles
25.	subgroup
26.	subgroups
27.	classification
28.	classifications
29.	17 or 18 or 19 or 20 or 21 or 22 or 23 or 24 or 25 or 26 or 27 or 28
30.	1 and 9 and 16 and 29

‘*’ indicates truncation.

NB: All search terms were adjusted for the requirements of the different databases. The time was limited from the year 2000 to 13 February 2018, but no other limitations were set concerning the document types or language with the search strategy.
