# Supplementary material for: Distinct trajectories of physical activity and related factors during the life course in the general population: a systematic review
Source: BMC Public Health. 2019 Mar 6;19:271. doi: 10.1186/s12889-019-6513-y (PMC6404287; doi:10.1186/s12889-019-6513-y)
Supplement: Supplementary file 3 — Table S3. Characteristics of the included studies, physical activity trajectories and related factors reported, and main findings in each study. (PDF 462 kb) [file 12889_2019_6513_MOESM3_ESM.pdf]

Additional file 3: Table S3. Characteristics of the included studies, physical activity trajectories and related factors reported, and main findings in each study.

Ref.	Aim of the study	Name of the study; geographical location; study design; year of the baseline measurement; follow-up time; number of measurements	Study sample; age at baseline	Finite mixture model used for identifying trajectories; software used; criteria used for model comparison, determining the final number of trajectory classes and goodness of fit of the model	Method for collecting PA data; PA variables used	Number and names of the identified PA trajectory classes, and the proportion of participants in each trajectory class	Determinants / predictors / confounders / mediators / outcomes / covariates of trajectory class membership	Main findings in relation to trajectory class membership
**Articles in the youngest group (children, adolescents and young adults)**								
Audrain-Mc-Govern et al. (2012)	1) Examine how variation in adolescent PA is associated with smoking and alternative tobacco use.	NR; USA; longitudinal study; NR; 4 years; 8	1429 adolescents (50% females, 73% White); 14 years	GGMM; Mplus; the proportion of cohort members in each class > 5%, BIC, ABIC, entropy*, BLRT	SR; MVPA was defined as sum score of hours / week in activities ≥ 3 METs	Five MVPA trajectories:<br>1) stable higher PA, 21%<br>2) decreased PA, 12%<br>3) stable regular PA, 15%<br>4) curvilinear PA, 5%<br>5) stable low PA, 47%	Outcomes:<br>smoking, and alternative tobacco use<br><br>Covariates:<br>gender, race, and parental education	Adolescents following the stable regular, stable low or decreasing PA trajectory had at least twice as great likelihood of smoking compared to those following the stable high PA trajectory. The prevalence of alternative tobacco use was the greatest among adolescents following decreased and stable regular trajectories at the age of 18 years. Adolescents whose PA significantly declined did not fall below the recommended level of PA but the decreasing PA trajectory had the greatest proportion of regular smokers and alternative tobacco users.
Farooq et al. (2017)	1) Identify when changes in PA take place during childhood to adolescence.	Gateshead Millenium Cohort Study; North-East England; longitudinal study; 2006–2007; 8 years; 4	545 children and adolescents (52% females); 7 years	GBTM; Stata Traj plugin; BIC, posterior probabilities, determining the final number of trajectories was stopped when substantial improvement in model fit was no more found	OBM; Total volume of PA and MVPA were measured using ActiGraph GT1M accelerometers (MVPA= 574 or more counts / 15 seconds)	One trajectory of total volume of PA, females:<br>1) decreasing, 100%<br><br>Three trajectories of total volume of PA, males:<br>1) high decreasing, 10%<br>2) moderate decreasing, 36%<br>3) low decreasing, 54%<br><br>Three trajectories of MVPA, females:	NA	Total PA volume declined already by age seven. It was suggested that greater emphasis should be put to PA promotion already in childhood.

						1) high initial MVPA with steeper declines, 19%<br>2) gradually declining, 62%<br>3) low and relatively stable with modest declines, 19%<br><br>Four trajectories of MVPA, males:<br>1) initially high and rapidly declining, 17%<br>2) relatively high and stable or increasing, 19%<br>3) gradually declining, 62%<br>4) low and relatively stable / slowly declining, 3%		
Findlay, Garner & Kohen (2009)	1) Identify distinct trajectories of organized PA participation among children and youth.<br>2) Explore the association of PA trajectories with potential risk factors.	National Longitudinal Survey of Children and Youth; Canada; longitudinal study; 1994; 8 years; 5	8817 children and adolescents (51% females); 4–11 years	Multiple trajectory modeling, semiparametric GBTM; SAS Proc Traj; BIC, posterior probabilities (majority of groups >0.7), all slope parameters had to be statistically significant	PR & SR; Organized PA participation was measured with two questions concerning the frequency of engagement to organized PA	Three trajectories of organized PA participation, females:<br>1) high decreasing participation, 37%<br>2) moderate stable participation, 24%<br>3) low decreasing participation, 39%<br><br>Three trajectories of organized PA participation, males:<br>1) high decreasing participation, 32%<br>2) low decreasing participation, 42%<br>3) high stable participation, 26%	Predictors: household income, parents' educational level, urban versus rural dwelling, and dual versus single parent at home	The decreasing trajectories of organized PA tended to peak in middle childhood (around 9 to 12 years of age) and then declined into adolescence. For boys and girls, higher parental education and income were associated with a greater likelihood of weekly participation in organized PA. Additionally, living in an urban area was significantly associated with a greater likelihood of weekly participation for girls.
Findlay, Garner & Kohen (2010)	1) Identify distinct trajectories of unorganized PA for children aged 4–17 years.<br>2) Study the association of PA trajectories with	National Longitudinal Survey of Children and Youth; Canada; longitudinal study; 1994; 8 years; 5	8978 children and adolescents (50% females); 4–11 years	Semiparametric GBTM; SAS Proc Traj; BIC, all slope parameters had to be statistically significant, posterior probabilities (>0.7)	PR & SR; Participation in unorganized PA was measured with one question concerning the frequency of participating in unorganized sports or PA outside of school	Two trajectories of unorganized PA, females:<br>1) regular participation, 57%<br>2) infrequent and decreasing participation, 43%	Predictors: household income, parents' educational level, single versus dual parent at home, and urban versus rural dwelling	Boys followed relatively stable trajectories of unorganized PA from childhood to adolescence while a group of decreasers was identified in girls. Out of the examined predictors, parents' higher educational level and having two parents at home predicted regular participation in unorganized PA, but only for boys.

	socioeconomic factors.					Two trajectories of unorganized PA, males:<br>1) regular participation, 69%<br>2) infrequent participation, 31%		
Howie et al. (2016)	1) Identify trajectories of organized SP from early childhood to late adolescence.<br>2) Explore the association of PA trajectories with physical and mental health outcomes in young adulthood.	Western Australian Pregnancy Cohort Study; Australia; longitudinal study; expecting mothers were recruited between years 1989-1991; 15 years; 6	1679 participants (49% females); 5 years	Repeated-measures LCA; LatentGOLD; BIC, AIC, BLRT, posterior probabilities (>0.7), entropy, classification errors, the percent of iterations converging on the same solution, meaningfulness of the solutions, odds of correct classification greater than five	PR; SP was measured with a question whether a child participated in organized sport outside of school hours (yes / no)	Three trajectories of organized SP, females:<br>1) consistent sport participators, 48%<br>2) sport dropouts, 34%<br>3) sport nonparticipators, 18%<br><br>Three trajectories of organized SP, males:<br>1) consistent sport participators, 55%<br>2) sport dropouts, 37%<br>3) sport joiners, 8%	Outcomes:<br>self-reported PA, body composition, self-rated health, self-rated well-being, mental health, mother’s and father’s race, and mother’s educational level at the age of 20 years<br><br>Covariate:<br>PA levels at the age of 20 years (total MET-minutes per week)	SP started to decrease at the end of 14 years in the consistent SP trajectories. A nonparticipation trajectory was identified only for girls, suggesting that girls probably should start SP before reaching the age of 8 years. Differences were found in health outcomes between trajectory classes, e.g. boys and girls with consistent SP had more preferable health outcomes, and boys with consistent SP had higher levels of PA at the age of 20 years. However, it is unknown whether SP caused improved health in young adulthood or whether SP was indicative of an overall positive health pattern.
Janz et al. (2014)	1) Identify trajectories of objectively measured MVPA from childhood to adolescence.<br>2) Study whether MVPA predicts bone strength.	Iowa Bone Develop-mental Study; USA; longitudinal study; 1998–2002; 15 years; 6	530 participants (50% females, 95% White); 5 years	LCA: SAS Proc Traj; BIC, individual-specific probabilities	OBM; MVPA was measured using ActiGraph accelerometers (MVPA = 2296 or more counts / min)	Three MVPA trajectories, females:<br>1) severely declining high activity, 6%<br>2) declining moderate activity, 45%<br>3) declining persistent inactivity, 50%<br><br>Three MVPA trajectories, males:<br>1) increasing high activity followed by a decline, 23%<br>2) increasing moderate activity followed by a decline, 37%<br>3) declining persistent inactivity, 40%	Outcome:<br>bone strength (dual X-ray energy absorptiometry and peripheral computer quantitative temography) measured at the age of 17 years<br><br>Covariates:<br>weight and height	The proportion of highly active children was very low, especially among girls. By the age 17 almost all girls, including girls from the once highly active group, were inactive. A persistently high PA level during childhood was significantly associated with greater bone strength among adolescent boys and girls even after reductions in PA level during adolescence.
Kwon, Lee & Carne-thon (2015)	1) Identify PA and television viewing trajectories among adolescent girls.<br>2) Demonstrate the risk factors (race) of PA trajectories.	National Growth and Health Study; USA; longitudinal study; 1987; 9 years; 7	2155 African-Americans (100% females, 52% Black); 9–10 years	LCGA, GBTM, dual-trajectory analyses; Stata Traj; BIC, trajectory shapes for similarity, odds of correct classification >5.0, the proportion of cohort members	SR; The sum of Habitual Activity Questionnaire scores for different activity categories (expressed as MET-times per week) was used as an indicator for the level of PA	Four PA trajectories, females:<br>1) substantially decreasing from high PA, 9%<br>2) maintaining moderate PA, 32%	Predictor:<br>race<br><br>Outcome:<br>television viewing	Eighty-eight per cent of the girls maintaining high PA level followed the decreasing television viewing trajectory, whereas 86% of the girls following the decreasing moderate PA trajectory also followed the increasing television viewing trajectory. A significantly lower proportion of Black girls followed the

	3) Study the relationship between PA and television viewing trajectories.			assigned in each class (needs to be close to the proportion estimated from the model), posterior probabilities (>0.7), interpretation of 99% confidence intervals		3) maintaining high PA, 6%<br>4) decreasing from moderate PA, 53%		maintenance PA trajectories than White girls.
Kwon et al. (2015a)	1) Identify distinct MVPA trajectories from 5 to 19 years.<br>2) Examine the associations of MVPA trajectories with SP and television viewing trajectories.	Iowa Bone Development Study; USA; longitudinal study; 1998–2002; 15 years; 7	537 participants (50% females, 95% White); 5 years	GBTM; Stata Traj; BIC, similarity of trajectory shapes, the proportion of participants in each class (needs to be close to the proportion estimated from the model), odds of correct classification >5.0, posterior probabilities (>0.7), interpretation of 99% confidence intervals	OBM for MVPA; MVPA was measured using ActiGraph accelerometers (MVPA = 2296 or more counts / min).<br><br>SR for SP; Participants who reported participating in at least one eligible sport were categorized as participating in organized sports (during the last six months at 1st measurement and during previous seven days at 2nd measurement).	Four MVPA trajectories:<br>1) consistently inactive, 15%<br>2) consistently active, 18%<br>3) decreasing moderate MVPA, 53%<br>4) substantially decreasing high MVPA, 14%<br><br>Three SP trajectories:<br>1) no SP, 14%<br>2) drop-out from SP, 40%<br>3) consistent SP, 46%	Outcomes:<br>SP and television viewing<br><br>Covariate:<br>sex	Four MVPA trajectories, three SP trajectories and four television viewing trajectories were identified. Especially girls' inactivity was persistent from childhood to adolescence. The participants following the consistently inactive trajectory also followed a trajectory of no SP. The consistently active trajectory was significantly associated with decreasing an already low television viewing time. SP could be one way to prevent a consistently inactive pattern.
Kwon et al. (2015b)	1) Study whether following certain MVPA trajectories during childhood and adolescence predicts different risk levels of becoming obese in young adulthood.	Iowa Bone Develop-mental Study; USA; longitudinal study; 1998–2002; 16 years; 7	493 participants (51 % females); 5 years	GBTM; Stata Traj; BIC, posterior probabilities (>0.7), the proportion of cohort members in each class, similarity of trajectory shapes, odds of correct classification (≥5.0), the proportion of a sample assigned to a certain group close to the proportion estimated from the model, interpretation of 99% confidence intervals	OBM; MVPA was measured using ActiGraph accelerometers (MVPA = 2296 or more counts / min).	Four MVPA trajectories (identified earlier in Kwon et al. 2015a):<br>1) consistently inactive, 15%<br>2) consistently moderately active, 18%<br>3) decreasing from a moderate level of MVPA, 53%<br>4) substantially decreasing high level of MVPA, 14%	Outcome:<br>obesity in young adulthood (percentage of body fat measured with whole body dual-energy X-ray absorptiometry)<br><br>Covariates:<br>sex, maternal education level, age of peak height velocity, and energy intake	Participants who were active as children but became less active with age were more likely to become obese in young adulthood when compared to consistently active participants.
Kwon et al. (2016)	1) Identify different latent classes of relationships among parental factors (family income,	Iowa Bone Development Study; USA; longitudinal study; 1998–2002; 15 years; 7	408 families, n=537 for MVPA trajectories (48% of children	GBTM; Stata Traj; BIC, posterior probabilities, similarity of trajectory shapes, proportion of cohort members in each class	OBM for MVPA; MVPA was measured using ActiGraph accelerometers (MVPA = 2296 or more counts / min).	Four MVPA trajectories (identified earlier in Kwon et al. 2015a):<br>1) consistently inactive, 15%	Predictors:<br>family support for PA, and variables of parental characteristics that correlate with child's PA behavior	More favorable PA and SP behaviors were observed among those in higher SES families and with higher PA engagement of parents. However, youth in the parental factor class described as having low family SES and regular PA in high school by the father tended to

	educational level, parents' regular PA in high school and adulthood).<br>2) Examine the influence of the parental classes on child's SP and MVPA trajectories.<br>3) Study if family support mediates the influence of parental patterns on child's SP and MVPA trajectories.		were females; 97% White); 5 years		SR for SP; Participants who reported participating in at least one eligible sport were categorized as participating in organized sports (during the last six months at 1st measurement and during previous seven days at 2nd measurement).	2) consistently active, 18%<br>3) decreasing moderate physical activity, 53%<br>4) substantially decreasing high physical activity, 14%<br><br>Three SP trajectories (identified earlier in Kwon et al. 2015a):<br>1) no SP, 14%<br>2) drop-out from SP, 40%<br>3) consistent SP, 46%	Covariate:<br>sex	follow the consistent SP trajectory. Thus, among lower SES families, the father's role may be important to promote youth to sustain SP. Family support was independently associated with participant's PA behavior, rather than mediating the influence of parental factors on child PA.
Rod-riguez & Audrain-Mc-Govern (2004)	1) Study the likelihood of smoking at 11th grade among adolescents following distinct trajectories of team sport participation.	NR; USA; longitudinal study; 2000; 2 years; 4	1098 high school students (52% females); 14 years	GGMM; Mplus; BIC, entropy	SR; Team participation was assessed on a 4-point scale describing the number of teams on which the participant played during the past 12 months	Four team participation trajectories:<br>1) erratic, 7%<br>2) decrease, 13%<br>3) high, 41%<br>4) low, 39%	Outcome:<br>smoking at 11th grade<br><br>Covariates:<br>gender, race, baseline smoking, baseline alcohol use, baseline PA, baseline extracurricular activity, baseline depressive symptoms	Adolescents following the decreasing and erratic team participation trajectories were more likely to smoke in grade 11 than individuals with high participation. Females were at high risk for following the low trajectory while non-whites were at high risk for following the decreasing and erratic trajectories.
**Articles in the middle group (mainly adults)**								
Barnett et al. (2008)	1) Identify LTPA trajectories.<br>2) Explore socioeconomic and demographic predictors of distinct LTPA trajectories.	Canada Fitness Survey 1981 / Campell's Survey of Well-being 1988 / Physical Activity Longitudinal Study 2002–2004; Canada; follow-up cohort study; 1981; 22 years; 3	884 adults (56% females); 18–60 years	LCGA, semi-parametric GBTM; SAS Proc Traj; BIC, assignment to the class with highest prior probability	SR; LTPA was measured with questions concerning frequency and duration of activities. Average daily energy expenditure was computed and expressed in kcal / kg / day.	Four LTPA trajectories:<br>1) inactive, 56%<br>2) increasers, 25%<br>3) active, 12%<br>4) decreasers, 7%	Predictors:<br>age, sex, degree of urbanization at baseline, highest reported education, and baseline family income	Socio-economic and demographic factors strongly predicted the probability of following certain LTPA trajectories. Older participants, females, those having lower household income, and with lower educational level were significantly less likely to follow active than inactive trajectories. Additionally, those having lower educational level and lower household income were significantly more likely to follow decreasing than active trajectory.
Dishman et al. (2010)	1) Identify trajectories of meeting a	NR; Hawaii; longitudinal	497 adults (64%	LCGA; Mplus; Vuong-Lo-Mendell Rubin likelihood ratio	SR; PA is expressed as MET-minutes / week. METs were used for	Four trajectories of meeting the recommendations for	Predictors:<br>initial baseline values and change in	Constructs of Transtheoretical model can be used for predicting possible changes in PA behavior. Those

	guideline for sufficient participation in health-promoting PA.<br>2) Model longitudinal change in constructs of the Transtheoretical Model.<br>3) Study the association of the changes in the constructs of the Transtheoretical Model with the PA trajectories.	study; NR; 2 years; 5	females; 32% Asian; 19% Native Hawaiian / Pacific Islander; 40% Caucasian; 8% Other); 18–90 years (mean = 49 years)	test, likelihood probability	categorizing people as meeting or not meeting the public health guidelines for sufficient regular PA.	participation in regular MVPA:<br>1) always met, 23%<br>2) not met to meeting, 19%<br>3) partially met - declining, 34%<br>4) never met, 24%	constructs of the transtheoretical model including experiential and behavioral processes<br><br>Covariates:<br>gender, age, race, education level, median annual household income, marital status, BMI	participants maintaining or attaining the PA guideline more likely retained higher scores in the variables of Transtheoretical model (except self-efficacy). Participants used experiential as well as behavioral processes when trying to increase or maintain their PA level.
Kaseva et al. (2016)	1) Identify distinct PA trajectories from childhood to adulthood.<br>2) Study the associations of PA with changes in depressive symptoms in adulthood.	Young Finns Study; Finland; longitudinal study; 1980; 31 years; 8	3596 at baseline, n=3564 for the PA trajectories (51% females); 9–18 years	LCGA; Mplus, and Stata; AIC, posterior probabilities (>0.7), practical consideration	SR; LTPA was measured with several questions (e.g., frequency and intensity of PA, participation in organized PA, participation in sport competitions, habitual way of spending leisure-time, frequency of vigorous PA, hours spent on vigorous PA, and average duration of PA session) between ages 9 and 49 years. LTPA index was created based on these questions.	Three trajectories of LTPA:<br>1) highly physically active, 4%<br>2) moderately physically active, 86%<br>3) lightly physically active, 10%	Outcome:<br>depressive symptoms in 2012<br><br>Covariates:<br>age, sex, BMI, participants' negative emotionality in childhood, parents' educational and income levels, participants' adulthood depressive symptoms (in 1992, 1997, 2001, and 2007), participants' educational and income levels, participants' experiences of social support, and smoking status	A decline in PA was seen in each activity trajectory. Higher PA level was associated with lower levels of depressive symptoms in adulthood when compared to lower PA level, however, the association disappeared when covariates were taken into account. Thus, PA from childhood to adulthood or PA in adulthood was not associated with the progression of depressive symptoms in adulthood.
Kim et al. (2016)	1) Identify distinct PA trajectories among women.<br>2) Study the correlates of PA trajectory class membership.	Women's Injury Study; Southwest Central region of USA; prospective cohort study; 2007–2008; 5 years (18	669 adults (100% females); ≥20 years (mean = 53 ± 13 years)	LCGA; SAS and Mplus; ABIC, entropy, posterior probabilities, Lo-Mendell-Rubin likelihood ratio test	OBM; PA was measured using Accusplit 120XL-xBX pedometers across 18 consecutive months. The participants reported the step-count data via web-based surveillance in 7- or 8-day intervals.	Three trajectories of PA, females:<br>1) active (10000–12499 steps / day), 12%<br>2) somewhat-active (7500–9999 steps / day), 41%<br>3) low-active (5000–7499 steps / day), 47%	Covariates:<br>age, race, marital status, family income, employment status, cardiovascular-related problems, bone-related problems, and percent of body fat	Relatively high proportion of women followed the low-active trajectory. Steps / day increased during spring and decreased during autumn and winter except in the active trajectory group where steps / day did not significantly decrease during autumn. Middle-aged (41–60 years) and older (>60 years) women, and obese or overweight women more likely followed the low-

		months for PA); weekly reporting of PA (median = 104 weeks in the studied sample)						active trajectory class than theactive classes. Women reporting having cardiovascular-related problems less likely followed the active trajectory.
Kivi-niemi et al. (2016)	1) Study the association between lifelong PA and cardiovascular autonomic function in midlife.	Northern Finland Birth Cohort 1966; Finland; longitudinal study; 1980; 34 years; 3	3062 participants (58% females); 14 years	LCA; NR; BIC, interpretability of the classification, conceptual meaningfulness of the model, sizes of the subgroups	SR; LTPA was measured with two questions concerning the frequency of participation in sports outside school hours (at the age of 14) and the frequency of participating to brisk PA / exercise during leisure-time (at the ages of 31 and 46) after which the two answers were combined for the LCA.	Three LTPA trajectories, females:<br>1) active, 23%<br>2) semiactive, 51%<br>3) inactive, 26%<br><br>Three LTPA trajectories, males:<br>1) active, 28%<br>2) semiactive, 43%<br>3) inactive, 29%	Outcome:<br>cardiovascular autonomic function (vagally mediated heart rate variability and cross-spectral baroreflex sensitivity) at the age of 46 years<br><br>Covariates:<br>smoking, alcohol consumption, sleep, sitting time, BMI, waist-to-hip ratio, blood pressure, lipid status, glucose status	Higher lifelong PA was independently associated with better cardiovascular autonomic function in midlife in women, while in men this association was mediated by other cardiometabolic and lifestyle factors.
Laddu et al. (2017a)	1) Identify distinct PA trajectories from young adulthood to middle age.<br>2) Study the association of PA trajectories with the prevalence of coronary artery calcification.	Coronary Artery Risk Development in Young Adults; USA; longitudinal study; 1985–1986; 25 years; 8	3175 (57% females); 18–30 years	GBTM; SAS Proc Traj; BIC, posterior probabilities, qualitative examination	SR; LTPA was measured with several questions (e.g., frequency of participation in vigorous or moderate intensity recreational sports, exercise, home maintenance, and occupational activities during the previous year). A total activity sum-score was expressed in exercise units where a threshold of 300 exercise units was determined as meeting PA guidelines.	Three PA trajectories:<br>1) three times PA guidelines, 8%<br>2) meeting PA guidelines, 35%<br>3) below PA guidelines, 57%	Outcome:<br>presence of coronary artery calcification<br><br>Covariates:<br>age, race, sex, hypertension, diabetes, smoking status, BMI, education, and hyperlipidemia	White participants following the ’three times PA guideline’ trajectory had higher odds of developing subclinical coronary artery disease by middle age compared to the participants following the ‘below PA guidelines’ trajectory.
Oura et al. (2016)	1) Study the association of LTPA trajectories from adolescence to middle age with vertebral dimensions in adulthood.	Northern Finland Birth Cohort 1966; Finland; longitudinal study; 1980; 34 years; 3	1188 participants (56% females); 14 years	LCA; NR; BIC, interpretability of the classification, conceptual meaningfulness, sizes of the subgroups	SR; LTPA was measured with two questions concerning the frequency of participation in sports outside school hours at the age of 14, and the frequency of participating to brisk PA / exercise during leisure-time at the ages of 31 and 46 years.	Three LTPA trajectories, females:<br>1) active, 24%<br>2) moderately active, 46%<br>3) inactive, 30%<br><br>Three LTPA trajectories, males:<br>1) active, 29%	Outcome:<br>vertebral dimensions (lumbar magnetic resonance imaging) at the mean age of 47 years<br><br>Covariates:<br>height, weight, BMI, SES (educational level), and smoking	A high level of lifetime LTPA was associated with greater vertebral size among women, but only to a small extent. No such association was observed among men.

						2) moderately active, 42%<br>3) inactive, 29%	status at the age of 46 years	
Rovio et al. (2017)	1) Identify distinct PA trajectories from childhood to midlife.<br>2) Identify determinants of the PA trajectory class membership.	Young Finns Study; Finland; longitudinal study; 1980; 31 years; 8	3596 at baseline, n=2841 for the PA trajectories (51% females); 9–18 years	GBTM; SAS Proc Traj; BIC, average posterior probabilities (>0.7)	SR; LTPA was measured with several questions (e.g., frequency and intensity of PA, participation in organized PA, participation in sport competitions, habitual way of spending leisure-time, frequency of vigorous PA, hours spent on vigorous PA, and average duration of PA session) between ages 9 and 49 years. LTPA index was calculated based on these questions.	Five trajectories of LTPA:<br>1) persistently active, 7%<br>2) decreasingly active, 14%<br>3) increasingly active, 14%<br>4) persistently low active, 51%<br>5) persistently inactive, 15%	Determinants: residential status, BMI, parental educational level in childhood, participants' self-reported educational level in adulthood, parental LTPA, participants' childhood academic performance, marital status, number of children, current smoking status, and alcohol drinking frequency	The prevalence of persistently low and inactive participants was high in the studied population. Academic achievement, education, having children, smoking, and alcohol use were the most prevailing determinants for the PA trajectories.
**Articles in the oldest group (late middle-aged and older adults)**								
Aggio et al. (2018)	1) Identify distinct PA trajectories from midlife to old age.<br>2) Identify predictors of PA trajectory class membership.	British Regional Heart Study; Great Britain; longitudinal study; 1978–1980; 20 years; 4	7735 adults (100% males), n=4952 for PA trajectories; 40–59 years	GBTM; Stata Traj plugin; BIC, log Bayes factor, the proportion of participants in each class at least 5%, close agreement between the estimated probability of group membership and actual proportion of the sample assigned to the group, posterior probabilities (>0.7), odds of correct classification based on posterior probabilities exceeding 5.	SR; PA level was studied by asking several questions concerning time spent on walking, recreational activities and engagement in sport / exercise. Responses to different type of PA were scored based on the intensity and frequency of the activity. A total PA index was created based on scores on type of PA (six categories).	Three PA trajectories, males:<br>1) low-decreasing, 25%<br>2) light stable, 51%<br>3) moderate-increasing, 24%	Predictors: occupation, marital status, number of children, health conditions, other health problems, BMI, smoking status, alcohol consumption, region of residence, and weekly breakfast cereal consumption<br><br>Covariates: cardiovascular disease diagnoses and employment status	A quarter of participants had persistently low PA level. The results showed that men who followed moderate increasing PA or light stable PA trajectories had fewer health conditions, had better social background characteristics in midlife and generally engaged in other healthy behaviors than men who followed low decreasing PA trajectory. Leaving employment (meaning usually retirement) and cardiovascular disease - related conditions varied according to trajectory class membership.
Artaud et al. (2016)	1) Identify separate trajectories of PA, smoking, alcohol, and fruit and vegetable consumption.<br>2) Study the associations between the trajectories and	Whitehall II cohort study; Great-Britain; longitudinal study; 1985–1988; 28 years (19 years for PA); 11 (health behaviors have been	6825 civil servants (71% males), n=10205 for PA trajectories; 35–55 years	GBTM; SAS Proc Traj; BIC, posterior probabilities (≥0.7)	SR; Frequency and duration of PA were categorized into recommended level, inactivity, and intermediate PA.	Four PA trajectories:<br>1) persistent inactivity, 15%<br>2) intermediate then inactivity, 36%<br>3) intermediate then recommended, 23%<br>4) persistently recommended level, 27%	Outcome: disability over 8 years (mobility, instrumental activities of daily living, and basic activities of daily living)<br><br>Covariates: sex, age, marital status, SES, disability	Forty-two per cent of the participants followed one unhealthy trajectory, 20% two or three, and 38% none. Higher education, being married or cohabiting, and a better health profile were associated with following fewer unhealthy trajectories. Unhealthy trajectories of PA, smoking, and alcohol consumption from midlife to old age were independently associated with increased risk of subsequent disability

	disability at older age.	assessed 5 times in years 1985–2004)					assessment, depressive symptoms, BMI, cognition, bone fractures, chronic conditions, psychotropic drug use, cardiovascular disease and its risk factors	with this risk increasing progressively with the number of unhealthy trajectories.
Gabriel et al. (2017)	1) Study the change in PA during midlife.<br>2) Study the association of PA trajectories with physical function in later life.	Study of Women's Health Across the Nation; USA; longitudinal study; 1996–1997; 20 years; baseline and 15 follow-ups (PA data were collected at baseline and at 6 follow-ups)	1771 adults (100% females); 42–52 years	LCGA; SAS Proc Traj; BIC, scientific plausibility, highest prior probability	SR; A sports index comprising of the intensity, frequency and duration of two most frequent sports, at least in moderate intensity level (≥ 3 METs), and sport and exercise activities over the previous 12 months	Five trajectories of PA, females:<br>1) highest, 14%<br>2) middle, 24%<br>3) decreasing, 22%<br>4) increasing, 13%<br>5) lowest, 26%	Outcome:<br>physical function (40 foot walk, 4 meter walk, repeated chair stands, and grip strength)<br><br>Covariates:<br>age, ethnicity, site, sociodemographic factors, other health behaviors (e.g., smoking status), BMI, self-rated health status, bodily pain, physical difficulties, menopausal status, hormone use, presence of depressive symptoms, and self-reported comorbidities	The highest PA trajectory group had the most favorable physical functioning outcomes (all $p < 0.001$) when compared to other PA groups. Statistically significant differences in the physical functioning were observed when all other trajectory groups were compared to the lowest or increasing PA trajectory group. Characteristics associated with the lowest PA trajectory group were being Hispanic and Black, being single or never married, having fair or poor overall health status, being obese, having income < $35,000 per year, being current cigarette smoker, having severe or very severe bodily pain, having reported physical difficulties and having osteoarthritis.
Hsu et al. (2013)	1) Identify separate trajectories of regular exercise, smoking, drinking alcohol, and having general health checkups for elderly men and women.<br>2) Identify multiple trajectories of four health-related behaviors.<br>3) Describe the longitudinal trajectories of multiple health behaviors and identify factors	Taiwan Longitudinal Survey on Aging; Taiwan; longitudinal study; 1996; 11 years; 4	5880 older people (99% community -dwelling), n=4800 for trajectories (47% females); ≥50 years	GBTM and joint trajectory model; SAS Proc Traj; BIC, parsimony principle	SR; Getting regular exercise was defined as exercising for at least 30 minutes three times per week.	Four trajectories of regular exercise, females:<br>1) none or little, 49 %<br>2) decreasing exercisers, 17%<br>3) increasing exercisers, 19%<br>4) regular exercisers, 14%<br><br>Four trajectories of regular exercise, males:<br>1) non-exercisers, 44%<br>2) decreasing exercisers, 11%<br>3) increasing exercisers, 23%<br>4) regular exercisers, 21 %	Predictors:<br>age, number of years of education, marital status, self-related health, depressive symptoms, social support, social participation, and economic satisfaction at baseline	Five distinct trajectory groups of multiple health-related behaviors were identified for men (smoking, inactive, healthy lifestyle, smoking and drinking, and quitting) and three for women (smoking and drinking, inactive and healthy lifestyle). Age, education, self-rated health, depressive symptoms, and economic satisfaction at baseline associated with the health behavior trajectories. Studying multiple longitudinal trajectories instead of only single behavior trajectories can give new insight concerning the clustering of health behaviors across time.

	related to the different trajectory groups.							
Laddu et al. (2017b)	1) Identify distinct trajectories of PA and body composition in older men.<br>2) Study the association of PA trajectories with body composition trajectories over time.	Osteoporotic Fractures in Men Study; USA; longitudinal study; 2000–2002; 7 years; 3	5964 adults (100% males); ≥64 years (mean = 74 years)	GBTM; Stata Traj; determining the maximum number of trajectory groups a priori, BIC, posterior probabilities, only specific groups comprised of at least 1% of the analytic cohort were included	SR; PA was measured with the Physical Activity Scale for the Elderly which measures total, occupational, household and LTPA over the previous seven days. A total PA score was summed for each participant.	Three PA trajectories, males:<br>1) high-declining, 7%<br>2) moderate-declining, 50%<br>3) low-declining, 43%	Outcome:<br>body composition	A decline in PA was observed in all PA trajectories, with the greatest decline in the high-declining PA trajectory group. Men in the high-declining group reported higher PA levels throughout the study period when compared to other two PA groups. Among high-declining and low-declining PA trajectory groups total body weight and lean mass significantly declined while fat mass stayed relatively unchanged. Fat mass increased in the moderate-declining PA trajectory group.
Nguyen et al. (2013)	1) Identify recreational PA trajectories among postmenopausal women<br>2) Examine whether women following different PA trajectories engage in different types of PA.<br>3) Study baseline sociodemographic characteristics, lifestyle behaviors, health, and psychosocial status as predictors of PA trajectory membership.	Women's Health Initiative Observation Study; USA; prospective, multicenter clinical trial and observational study; 1993–1998; 8 years; 7	92629 adults (100% females, 83% White); 50–79 years women were divided into three age-groups at baseline: 50–59 years (32%), 60–69 years (44%) and >70 years (24%).	Latent profile analyses, latent growth curve modeling; Mplus; BIC, AIC, class interpretability, class prevalence, entropy (higher value close to 1 representing a better classification)	SR; Recreational PA was defined as energy expenditure expressed as MET-hours / week.	Three recreational PA trajectories across the three age groups (50–59 / 60–69 / >70 years), females:<br>1) minimally active, ranging between 70%–74% in each age group<br>2) moderately active, 23%–25%<br>3) highly active, 4%–5%	Predictors:<br>age, ethnicity, marital status, living situation, education, income, employment status, caregiving responsibilities, current residence, smoking, dietary patterns, alcohol intake, hormone use, previous participation in vigorous exercise, height, weight, health-related quality of life, history of falls, hip fractures, hospitalizations, chronic medical illnesses, depressive symptoms, history of depression, and social support	Nearly 75% of the participants were following the minimally active trajectory. Sociodemographic characteristics (e.g., ethnicity, income, and education), some health status indicators (e.g., BMI), and past vigorous PA were predictive of PA trajectory class membership.
Pan et al. (2015)	1) Identify PA trajectories among Taiwanese older adults.<br>2) Identify the related factors of the different trajectories.	Taiwan Longitudinal Study for Aging; Taiwan; follow-up study; 1996; 11 years; 4	4018 older adults (49% females); 50–96 years (mean = 65 years)	GBTM; SAS Proc Traj; BIC, posterior probabilities (≥0.7)	SR; Frequency and duration of sports and exercise. Being physically active was determined as performing sports or exercises at least three times in a week, and at least for 30 minutes each time.	Four PA trajectories:<br>1) inactive, 48%<br>2) decreasing, 12%<br>3) increasing, 23%<br>4) active, 17%	Covariates:<br>sex, years of education, age, ethnicity, depressive symptoms, physical functional limitations, self-rated health, marital status, economic satisfaction, residential area, and working status	Heterogeneity of PA was found among Taiwanese older adults. Nearly half of the participants were following the inactive trajectory. Older age and higher educational level were positively associated with being active. Those participants having jobs, depressive symptoms, and several physical functional limitations were less likely to be physically active in the decreasing, increasing, and active patterns.

Xue et al. (2012)	1) Identify PA trajectories among older women.<br>2) Assess the associations of PA trajectories with all-cause mortality.<br>3) Identify predictors of change in PA.	Women's Health and Aging Study II; USA; prospective cohort study; 1994; 12 years; 7	433 adults (100% females); 70–79 years	Joint latent class and survival mixture model; Mplus, and Stata; Lo-Mendell-Rubin adjusted likelihood ratio test, scientific plausibility, meaningfulness of the trajectory patterns, person-specific probabilities (higher value representing a better classification)	SR; The frequency and duration of participation into different exercise, and lifestyle and outdoor activities. Activity level was classified into three categories (inactive, moderately active and very active) based on kcal / kg / day.	Four PA trajectories, females:<br>1) always active, 17%<br>2) fast declining, 19%<br>3) stable moderate, 32%<br>4) always sedentary, 32%	Predictors:<br>BMI, number of chronic diseases, having depressive symptoms, mobility disability, living alone, self-efficacy, energy level, self-reported health, and smoking status<br><br>Outcome:<br>all-cause mortality<br><br>Covariates:<br>baseline age, race, and educational level	Maintaining an active or moderately active lifestyle was associated with the lowest mortality rate. A curvilinear relationship between the lower levels of PA and higher mortality risk was observed indicating that the greatest gain of increasing PA would be among those women with lowest PA levels. Depressive symptoms, coronary artery disease, mobility disability, obesity, chronic obstructive pulmonary disease, low self-efficacy, and low energy were associated with sedentary behavior and with a fast decline in activity.

NR not reported; *NA* not applicable; *PA* physical activity; *SP* sport participation; *MVPA* moderate to vigorous intensity physical activity; *LTPA* leisure-time physical activity; *MET* metabolic equivalent; *SR* self-report; *PR* parent-report; *OBM* objectively measured; *GBTM* group-based trajectory modeling; *GGMM* general growth mixture modeling; *LCA* latent class analysis; *LCGA* latent class growth analysis; *BLRT* bootstrap likelihood ratio test; *BIC* Bayesian information criterion; *ABIC* adjusted Bayesian information criterion; *AIC* Akaike's information criterion; *BMI* body mass index; *SES* socioeconomic status

**Entropy* = Average classification probability
