# Supplementary material for: Distinct trajectories of physical activity and related factors during the life course in the general population: a systematic review
Source: BMC Public Health. 2019 Mar 6;19:271. doi: 10.1186/s12889-019-6513-y (PMC6404287; doi:10.1186/s12889-019-6513-y)
Supplement: Supplementary file 4 — Forest plot. Physical activity trajectories divided into categories by age group. (PDF 264 kb) [file 12889_2019_6513_MOESM4_ESM.pdf]

A) From increaser to decreaser / Increaser **

	N	Measure	Method	Class size (SE)
**Youngest group**				
1 Audrain−McGovern, 2012	1,429	MVPA	SR	0.05 (0.01)
2 Farooq 2017: Females	283	Total PA volume	OBM	
3 Farooq 2017: Males	262	Total PA volume	OBM	
4 Farooq 2017: Females	283	MVPA	OBM	
5 Farooq 2017: Males	262	MVPA	OBM	
6 Findlay, 2009: Females	4,497	Organized PA	PR + SR	
7 Findlay, 2009: Males	4,320	Organized PA	PR + SR	
8 Findlay, 2010: Females	4,489	Unorganized PA	PR + SR	
9 Findlay, 2010: Males	4,489	Unorganized PA	PR + SR	
10 Howie, 2016: Females	823	Organized SP	PR	
11 Howie, 2016: Males	856	Organized SP	PR	0.08 (0.01)
12 Janz, 2014: Females	265	MVPA	OBM	
13 Janz, 2014: Males	265	MVPA	OBM	0.23 (0.03)
14 Kwon, 2015a*	537	MVPA	OBM	
15 Kwon, 2015a*	537	SP	SR	
16 Kwon, Lee, Carnethon, 2015: Females	2,155	PA	SR	
17 Rodriguez, 2004	1,098	Team participation	SR	0.07 (0.01)
**Middle group**				
18 Barnett, 2008	884	LTPA	SR	0.25 (0.01)
19 Dishman, 2010	497	MVPA	SR	0.19 (0.02)
20 Kaseva, 2016	3,564	LTPA	SR	
21 Kim, 2016: Females	669	PA	OBM	
22 Kiviniemi*, 2016: Females	1,776	LTPA	SR	
23 Kiviniemi*, 2016: Males	1,286	LTPA	SR	
24 Laddu, 2017a	3,175	LTPA	SR	
25 Rovio, 2017	2,841	LTPA	SR	0.14 (0.01)
**Oldest group**				
26 Aggio, 2018: Males	4,952	PA	SR	0.24 (0.01)
27 Artaud, 2016	10,205	PA	SR	0.23 (0.00)
28 Gabriel, 2017: Females	1,771	Sport and exercise	SR	0.13 (0.01)
29 Hsu, 2013: Females	2,256	Regular exercise	SR	0.19 (0.01)
30 Hsu, 2013: Males	2,544	Regular exercise	SR	0.23 (0.01)
31 Laddu, 2017b: Males	5,964	PA	SR	
32 Nguyen, 2013: Females	92,629	PA	SR	
33 Pan, 2015	4,018	PA	SR	0.23 (0.01)
34 Xue, 2012: Females	433	PA	SR	

Class size (95% CI)

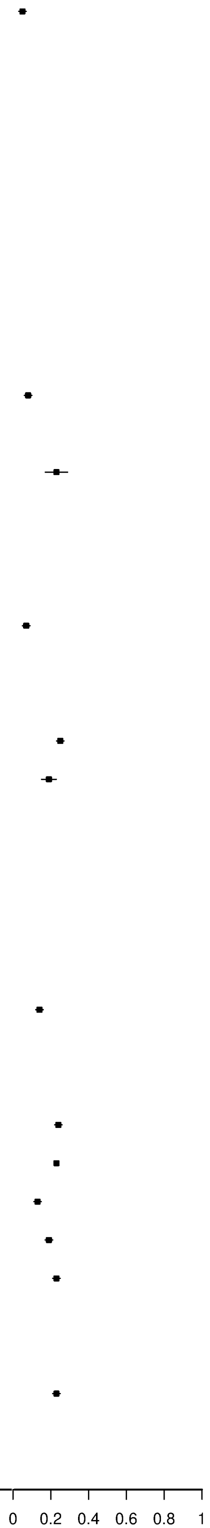

	B) Highly active Class size (SE)	C) Active Class size (SE)	D) Inactive Class size (SE)
**Youngest**			
1	0.21 (0.01)	0.15 (0.01)	
2			
3			
4			
5		0.19 (0.02)	
6		0.24 (0.01)	
7	0.26 (0.01)		
8		0.57 (0.01)	
9		0.69 (0.01)	
10		0.48 (0.02)	0.18 (0.01)
11		0.55 (0.02)	
12			0.50 (0.03)
13			0.40 (0.03)
14		0.18 (0.02)	0.15 (0.02)
15		0.46 (0.02)	0.14 (0.01)
16	0.06 (0.01)	0.32 (0.01)	
17		0.41 (0.01)	
**Middle**			
18		0.12 (0.01)	0.56 (0.02)
19		0.23 (0.02)	0.24 (0.02)
20	0.04 (0.00)	0.86 (0.01)	
21	0.12 (0.01)	0.41 (0.02)	
22	0.23 (0.01)	0.51 (0.01)	0.26 (0.01)
23	0.28 (0.01)	0.43 (0.01)	0.29 (0.01)
24	0.08 (0.00)	0.35 (0.01)	
25		0.07 (0.00)	0.15 (0.01)
**Oldest**			
26		0.51 (0.01)	
27		0.27 (0.00)	0.15 (0.00)
28	0.14 (0.01)	0.24 (0.01)	
29		0.14 (0.01)	0.49 (0.01)
30		0.21 (0.01)	0.44 (0.01)
31	0.07 (0.00)		
32	0.04 (0.00)	0.24 (0.00)	
33		0.17 (0.01)	0.48 (0.01)
34	0.17 (0.02)	0.32 (0.02)	0.32 (0.02)

Class size (95% CI) axis: 0 0.2 0.4 0.6 0.8 1

	E) Decreaser of high PA Class size (SE)	F) Decreaser of moderate PA Class size (SE)	G) Decreaser of low PA / Low-active Class size (SE)
**Youngest**			
1	0.12 (0.01)		0.47 (0.01)
2		1.00 (0.00)	
3	0.10 (0.02)	0.36 (0.03)	0.54 (0.03)
4	0.19 (0.02)	0.62 (0.03)	0.19 (0.02)
5	0.17 (0.02)	0.62 (0.03)	0.03 (0.01)
6	0.37 (0.01)		0.39 (0.01)
7	0.32 (0.01)		0.42 (0.01)
8			0.43 (0.01)
9			0.31 (0.01)
10		0.34 (0.02)	
11		0.37 (0.02)	
12	0.06 (0.01)	0.45 (0.03)	
13		0.37 (0.03)	
14	0.14 (0.01)	0.53 (0.02)	
15		0.40 (0.02)	
16	0.09 (0.01)	0.53 (0.01)	
17		0.13 (0.01)	0.39 (0.01)
**Middle**			
18		0.07 (0.01)	
19		0.34 (0.02)	
20			0.10 (0.01)
21			0.47 (0.02)
22			
23			
24			0.57 (0.01)
25		0.14 (0.01)	0.51 (0.01)
**Oldest**			
26			0.25 (0.01)
27		0.36 (0.00)	
28		0.22 (0.01)	0.26 (0.01)
29		0.17 (0.01)	
30		0.11 (0.01)	
31		0.50 (0.01)	0.43 (0.01)
32			0.72 (0.00)
33		0.12 (0.01)	
34		0.19 (0.02)	

Class size (95% CI): 0 0.2 0.4 0.6 0.8 1

Abbreviations: N = sample size of the study population; SE = standard error; CI = confidence interval; PA = physical activity; MVPA = moderate to vigorous intensity physical activity; LTPA = leisure-time physical activity; SP = sport participation; OBM = objectively measured; SR = self-reported; PR = parent-reported.

*These exact same trajectories were reported in other study / other studies as well. The duplicates were omitted from the figure.

**The From increaser to decreaser category applies to the youngest group while the Increaser category applies to the middle and the oldest group.
