# Supplementary material for: Distinct trajectories of physical activity and related factors during the life course in the general population: a systematic review
Source: BMC Public Health. 2019 Mar 6;19:271. doi: 10.1186/s12889-019-6513-y (PMC6404287; doi:10.1186/s12889-019-6513-y)
Supplement: Supplementary file 5 — Table S4. Quality assessment checklist and quality scores of the included studies. (PDF 180 kb) [file 12889_2019_6513_MOESM5_ESM.pdf]

Additional file 5: Table S4. Quality assessment checklist and quality scores of the included studies.

	Questions (1-9) modified from the Quality Assessment Tool for Observational Cohort and Cross-Sectional Studies									Questions (11-16) modified from the Guidelines for Reporting on Latent Trajectory Studies							
Author (publication year)	1. Was the research question or objective in this paper clearly stated?	2. Was the study population specified and defined?	3. Was the participation rate of eligible persons at least 50% at baseline?	4. Was loss to follow-up after baseline defined?	5. Were inclusion and exclusion criteria for the selected subjects defined?	6. Were the potential covariates, confounders, determinants and / or predictors defined?	7. Were the PA[a] measures clearly defined, and implemented consistently across all study participants?	8. Was the validity and / or reliability of the PA measures reported?	9. Were the PA measures assessed at least three times (i.e., the minimum number of measurement points needed when identifying trajectories)?	10. Was PA objectively measured?	11. Was more than one statistical fit measure taken under consideration when deciding the final number of trajectory classes and was at least one of the used fit measures BIC or ABIC?	12. Were the values of the model fit measures (e.g., BIC, ABIC or AIC) concerning the final solution reported?	13. Were posterior probability values and / or entropy values of the final solution reported or their cut-off values defined?	14. Was a plot of the final solution with the estimated mean trajectories provided?	15. Were the total number of fitted models clearly reported?	16. Were the values of the model fit measures of all the fitted models clearly reported?	**Sum quality score[b] (range 0-16)**	**Quality of the study[c]**
**YOUNGEST GROUP:**																	
Audrain-McGovern et al. (2012)	Yes	Yes	Yes	Yes	Yes	Yes	Yes	Yes	Yes	No	Yes	Yes	Yes	Yes	Yes	Yes	15	good
Farooq et al. (2017)	Yes	Yes	Yes	Yes	Yes	NA	Yes	Yes	Yes	Yes	Yes	No	No	Yes	No	No	11	fair
Findlay, Garner & Cohen (2009)	Yes	Yes	CA	CA	Yes	Yes	Yes	Yes	Yes	No	Yes	No	No	Yes	No	No	9	fair

Findlay, Garner & Cohen (2010)	Yes	Yes	Yes	Yes	Yes	Yes	Yes	Yes	Yes	No	Yes	No	Yes	Yes	No	No	12	good
Howie et al. (2016)	Yes	Yes	CA	Yes	Yes	Yes	Yes	Yes	Yes	No	Yes	Yes	Yes	Yes	Yes	Yes	14	good
Janz et al. (2014)	Yes	Yes	CA	Yes	Yes	Yes	Yes	Yes	Yes	Yes	Yes	No	No	Yes	No	No	11	fair
Kwon, Lee & Carnethon (2015)	Yes	Yes	Yes	Yes	Yes	Yes	Yes	Yes	Yes	No	Yes	Yes	Yes	Yes	Yes	Yes	15	good
Kwon et al. (2015a)	Yes	Yes	CA	Yes	Yes	Yes	Yes	Yes	Yes	Yes	Yes	Yes	Yes	Yes	Yes	Yes	15	good
Kwon et al. (2015b)	Yes	Yes	CA	Yes	Yes	Yes	Yes	Yes	Yes	Yes	Yes	Yes	Yes	Yes	Yes	Yes	15	good
Kwon et al. (2016)	Yes	Yes	CA	Yes	Yes	Yes	Yes	Yes	Yes	Yes	Yes	Yes	Yes	Yes	Yes	Yes	15	good
Rodriguez & Audrain-McGovern (2004)	Yes	Yes	Yes	No	Yes	Yes	Yes	No	Yes	No	Yes	Yes	Yes	Yes	Yes	Yes	13	good
**MIDDLE GROUP:**																		
Barnett et al. (2008)	Yes	Yes	Yes	Yes	Yes	Yes	Yes	Yes	Yes	No	Yes	No	Yes	Yes	No	No	12	good
Dishman et al. (2010)	Yes	Yes	Yes	Yes	Yes	Yes	Yes	Yes	Yes	No	No	No	Yes	Yes	No	No	11	fair
Kaseva et al. (2016)	Yes	Yes	Yes	Yes	Yes	Yes	Yes	Yes	Yes	No	No	Yes	Yes	Yes	Yes	Yes	14	good
Kim et al. (2016)	Yes	Yes	Yes	Yes	Yes	Yes	Yes	Yes	Yes	Yes	Yes	Yes	Yes	No	Yes	Yes	15	good
Kiviniemi et al. (2016)	Yes	Yes	Yes	Yes	Yes	Yes	Yes	No	Yes	No	No	No	No	NA	Yes	No	9	fair
Laddu et al. (2017a)	Yes	Yes	Yes	Yes	Yes	Yes	Yes	Yes	Yes	No	Yes	No	Yes	Yes	No	No	12	good
Oura et al. (2016)	Yes	Yes	Yes[d]	Yes	Yes	Yes	Yes	No	Yes	No	No	No	No	NA	Yes	No	9	fair
Rovio et al. (2017)	Yes	Yes	Yes	Yes	Yes	Yes	Yes	Yes	Yes	No	Yes	No	Yes	Yes	Yes	No	13	good
**OLDEST GROUP:**																		
Aggio et al. (2018)	Yes	Yes	Yes	Yes	Yes	Yes	Yes	Yes	Yes	No	Yes	Yes	Yes	Yes	Yes	Yes	15	good
Artaud et al. (2016)	Yes	Yes	Yes	Yes	Yes	Yes	Yes	No	Yes	No	Yes	No	Yes	Yes	No	No	11	fair

Gabriel et al. (2017)	Yes	Yes	Yes	Yes	Yes	Yes	Yes	Yes	Yes	No	Yes	No	No	Yes	No	No	11	fair
Hsu et al. (2013)	Yes	Yes	Yes	Yes	Yes	Yes	Yes	No	Yes	No	No	Yes	No	Yes	No	No	10	fair
Laddu et al. (2017b)	Yes	Yes	Yes	Yes	Yes	NA	Yes	Yes	Yes	No	Yes	No	No	Yes	No	No	10	fair
Nguyen et al. (2013)	Yes	Yes	Yes	Yes	Yes	Yes	Yes	Yes	Yes	No	Yes	Yes	Yes	Yes	Yes	Yes	15	good
Pan et al. (2015)	Yes	Yes	Yes	Yes	Yes	Yes	Yes	No	Yes	No	Yes	Yes	Yes	Yes	Yes	Yes	14	good
Xue et al. (2012)	Yes	Yes	Yes	Yes	Yes	Yes	Yes	Yes	Yes	No	No	No	No	Yes	No	No	10	fair

PA physical activity; *BIC* Bayesian information criterion; *ABIC* adjusted Bayesian information criterion; *NA* not applicable; *CA* can’t answer

[a]PA includes also sport participation and exercise variables.

[b]1 when the criterion was applicable to the analyzed study (1 = yes); 0 when the criterion was not fulfilled, not applicable, or could not be answered based on the information provided by the study (0 = no).

[c]Poor (score ranging from 0 to 5); fair (score ranging from 6 to 11); good (score ranging from 12 to 16).

[d]the participation rate was less than 50% from the original sample but it was 77% of those who had undergone lumbar magnetic resonance imaging.
